# Roles and Mechanisms of the Long Noncoding RNAs in Cervical Cancer

**DOI:** 10.3390/ijms21249742

**Published:** 2020-12-21

**Authors:** Miguel Ángel Cáceres-Durán, Ândrea Ribeiro-dos-Santos, Amanda Ferreira Vidal

**Affiliations:** 1 Laboratory of Human and Medical Genetics, Institute of Biological Sciences, Graduate Program of Genetics and Molecular Biology, Federal University of Pará, Belém 66075-110, Brazil; macdur@gmail.com (M.Á.C.-D.); akelyufpa@gmail.com (Â.R.-d.-S.); 2 Graduate Program in Oncology and Medical Sciences, Center of Oncology Researches, Federal University of Pará, Belém 66073-005, Brazil

**Keywords:** cervical cancer, epigenetics, long noncoding RNAs, biological function, tumorigenesis

## Abstract

Cervical cancer (CC) continues to be one of the leading causes of death for women across the world. Although it has been determined that papillomavirus infection is one of the main causes of the etiology of the disease, genetic and epigenetic factors are also required for its progression. Among the epigenetic factors are included the long noncoding RNAs (lncRNAs), transcripts of more than 200 nucleotides (nt) that generally do not code for proteins and have been associated with diverse functions such as the regulation of transcription, translation, RNA metabolism, as well as stem cell maintenance and differentiation, cell autophagy and apoptosis. Recently, studies have begun to characterize the aberrant regulation of lncRNAs in CC cells and tissues, including Homeobox transcript antisense RNA (HOTAIR), H19, Metastasis-associated lung adenocarcinoma transcript 1 (MALAT1), Cervical Carcinoma High-Expressed 1 (CCHE1), Antisense noncoding RNA in the inhibitors of cyclin-dependent kinase 4 (ANRIL), Growth arrest special 5 (GAS5) and Plasmacytoma variant translocation 1 (PVT1). They have been associated with several disease-related processes such as cell growth, cell proliferation, cell survival, metastasis and invasion as well as therapeutic resistance, and are novel potential biomarkers for diagnosis and prognosis in CC. In this review, we summarize the current literature regarding the knowledge we have about the roles and mechanisms of the lncRNAs in cervical neoplasia.

## 1. Introduction

Cervical cancer (CC) is the fourth most frequent cancer in women globally. It is a preventable disease and also curable if detected early and adequately treated. Yet it remains one of the most common cancers and the leading cause of cancer-related death in women across the globe [1].

CC occurs at the epithelium of the uterine cervix, particularly at the squamocolumnar junction of the ectocervix and endocervix, a site of continuous metaplastic activity [2]. Nearly all CCs are caused by human papillomavirus (HPV) infections, which is the most common sexually transmitted infection worldwide [3].

The two major histological types of epithelial tumors in the cervix are squamous cell carcinoma (SCC) and adenocarcinoma, the first being the most common one, comprising 95% of all diagnosed CCs worldwide, while the remaining 5% is represented by adenocarcinoma and other less common epithelial tumors [2].

The most common therapies used for CC treatment are radiotherapy and chemotherapy, but these methods are not always effective and can cause severe side effects. Hence, there is an evident interest in finding new biomarkers and novel treatment targets that could complement standard evaluation to determine the presence of cancer cells in cervical tissues [4,5].

Integration of high-risk HPV (HR-HPV) viral DNA into the host chromosomal DNA is the main pathological event involved in CC. HPV DNA is the only molecular marker developed for CC diagnosis. For this reason, it is necessary to evaluate other events that could be used as possible markers based on their clinical utility, such as chromosomal anomalies, cell-cycle checkpoints, DNA mutations, epigenetic regulation, among others [5].

There are some markers, such as P16, P16^INK4A^, P53, RB, E-cadherin, and Ki67, that Z showed the ability to be able to detect intraepithelial lesions that can evolve to invasive forms. Additionally, markers such as CEA, SCC-Ag and CD44 can detect invasive forms of the disease. Overall, several studies have been focused on detecting biomarkers capable of identifying molecular changes that lead to the development and progress of CC [6].

Many noncoding RNAs (ncRNAs) were identified as molecular regulatory factors in cancer and may provide therapeutic targets for improving survival in cases of CC [4]. In recent years, it has been estimated that around 99% of the total RNA present in mammalian cells is comprised of ncRNA. Currently, it is not possible to know the exact amount of truly functional ncRNAs, as the number of these transcripts grows annually [7].

Based on their functions, the ncRNAs can be divided into two broad classes: housekeeping ncRNAs and regulatory ncRNAs. Housekeeping ncRNAs mainly regulate generic cellular functions—for instance, messenger RNA (mRNA) translation, splicing, and rRNA modification. On the other hand, regulatory ncRNAs can be divided, based on the length of the transcript, into short noncoding transcripts comprising less than 200 nucleotides (nt) and long noncoding RNAs comprising transcripts greater than 200 nt. [8]. This class of non-protein-coding RNA and functional RNA molecules include long noncoding RNAs (lncRNAs), microRNAs (miRNAs), small interfering RNA (siRNA), small nucleolar RNAs (snoRNA), piwi-interacting RNA (piRNA), among others [9,10].

The regulatory ncRNAs play critical roles in important biological processes such as DNA transcription, and mRNA post-transcriptional processing and translation. Furthermore, ncRNA can be packaged into exosomes and other extracellular vesicles, providing a mechanism for intercellular communication. Overwhelming evidence suggests that various ncRNAs can be implicated in human disease processes [9,10].

MiRNAs can target, by base-pairing, multiple mRNAs and modulate their expression. Many studies have reported the association of miRNAs and CC progression, involving mechanisms that affect apoptosis, cell proliferation, migration and invasion. Dysregulation of miRNAs at different stages of CC can play an important role in the development of the disease, as they are functionally involved in cell-cycle, P53, and Wnt signaling pathways, among others. Thus, stage-specific miRNAs could be used as CC biomarkers [11].

In addition to the role of the miRNAs in CC, other molecules have gained interest in oncology, since they are dysregulated in this pathology, including the lncRNAs. Accordingly, in this review, we present an updated version of the molecular mechanisms involved with some lncRNAs that play critical roles in CC. We focus on the interplay between lncRNAs and miRNAs, and the importance of such interactions during the tumorigenic process as well as signaling pathways, molecules and events involved in the disease.

## 2. Long Noncoding RNAs (lncRNAs)

LncRNAs, defined as transcripts of more than 200 nt that generally do not code for proteins, have been associated with diverse functions and represent the largest class of ncRNAs. In contrast to short ncRNAs, which are mostly attributed to gene regulation, the mechanistic role of lncRNAs is highly diverse, increasing their functional complexity [6]. LncRNAs have been classified according to their relative location into sense lncRNAs, antisense lncRNAs, bidirectional lncRNAs, intron lncRNAs, intergenic lncRNAs and enhancer lncRNAs [12].

**Figure 1:**
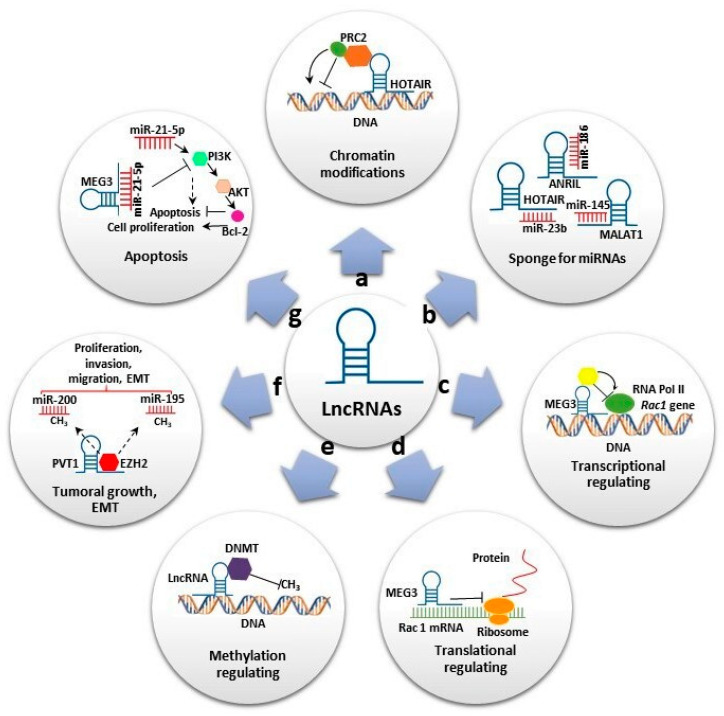
Long noncoding RNAs’ (lncRNAs) biological roles. (**a**) Some lncRNAs regulate gene expression by assembling chromatin-modifying complexes. Homeobox transcript antisense RNA (HOTAIR) interacts with polycomb repressive complex 2 (PRC2) and establishes the repressive H3K27me3 chromatin mark. (**b**) lncRNAs can act as miRNA sponges. HOTAIR, Metastasis-associated lung adenocarcinoma transcript 1 (MALAT1) and Antisense noncoding RNA in the inhibitors of cyclin-dependent kinase 4 (ANRIL) sponge miR-23-b, miR-154 and miR-186, respectively, to reverse suppression of their target genes. (**c**,**d**) Some lncRNAs promote or suppress gene expression. Maternally expressed gene 3 (MEG3) inhibits *Rac1* at both transcriptional and translational levels. (**e**) Some lncRNAs interact with diverse DNMT members, promoting or repressing DNA methylation. (**f**) Some lncRNAs play important roles in cell migration, proliferation and invasion. Plasmacytoma variant translocation 1 (PVT1) binds to EZH2, increasing H3K27me3 level on miR-195 and miR-200b promoters and promoting epithelial–mesenchymal transition (EMT). (**g**) lncRNAs also play significant roles in apoptosis. MEG3 reduce the level of miR-21-5p expression, inhibiting cell proliferation and increasing apoptosis by regulating the PI3K/AKT/BCL-2/Bax/P21 pathway.

They participate in several biological processes at transcriptional, post-transcriptional and epigenetic levels (Figure 1). At the transcriptional level, lncRNAs induce or suppress gene expression by inducing chromatin modifications and or interacting with RNA polymerase, acting as miRNA sponges (Figure 1a,c). At the post-transcriptional level, lncRNAs can act as sponge for miRNAs (Figure 1b) and also can form a stable structure of double-stranded RNA with mRNAs and regulate the translation (Figure 1d). Additionally, they can bind to proteins and regulate their stability (Figure 1d).

At the epigenetic level, the lncRNAs are involved in various mechanisms, including gene silencing through DNA methylation and reconstruction of chromatin conformation by acetylation, methylation or ubiquination of histones. (Figure 1e) [13]. In addition, some of these transcripts have been shown to play important roles in stem cell maintenance and differentiation, cell autophagy, apoptosis and embryonic development (Figure 1f,g). Furthermore, lncRNAs were also reported as having oncogenic functions in many types of cancers and neurological and cardiovascular diseases [14].

## 3. LncRNAs Dysregulated in Cancer Cervical

**Figure 2:**
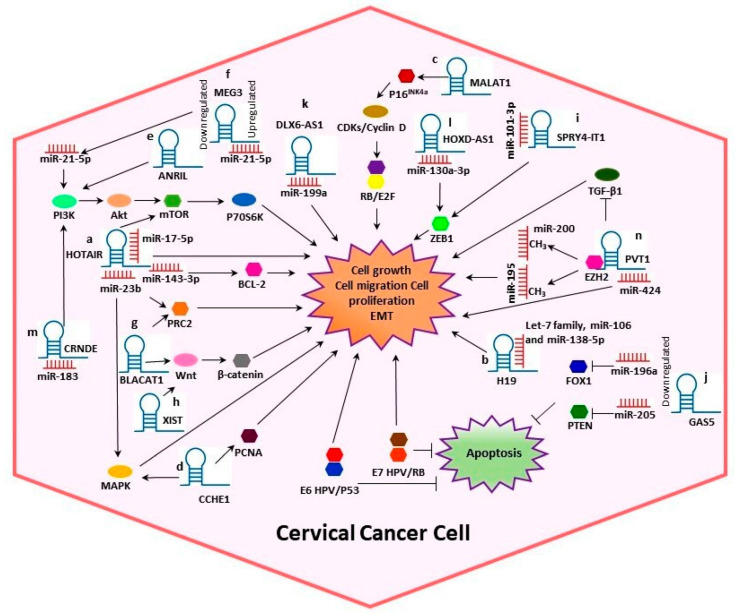
Potential mechanisms of several lncRNAs in CC. Some lncRNAs play significant roles in CC progression by sponging miRNAs and interacting with other proteins, altering the cell cycle. (**a**) HOTAIR could act as a sponge for miR-143-3p, promoting *BCL-2* expression. Also, HOTAIR exerts its tumor-promoting effect by sponging miR-17-5p and may indirectly modulate MAPK1 expression by binding to miR-23b. (**b**) H19 interacts with miRNAs of the let-7 family regulating diverse cellular processes. (**c**) MALAT1 may regulate cell proliferation through the P16^INK4A^/CDKs/RB pathway. (**d**) Cervical Carcinoma High-Expressed 1 (CCHE1) enhancing the expression of PCNA, CCHE1 also participates in the ERK/MAPK pathway. (**e**) ANRIL inhibition guides the inactivation of the PI3K/Akt pathway. (**f**) Maternally expressed gene 3 (MEG3) functions as a tumor-suppressor via regulating miR-21-5p. (**g**) Bladder cancer-associated transcript 1 (BLACAT1) binds to PRC2 and promotes proliferation, migration and invasion by modulating Wnt/β-catenin pathway. (**h**) X-inactive specific transcript (XIST) can play the role of an oncogene activating the Wnt/β-catenin pathway. (**i**) SPRY4-Intronic transcript or Sprouty4-Intronic transcript 1 (SPRY4-IT1) could bind to miR-101-3p to regulate the expression of the target gene ZEB1. (**j**) GAS5 expression is decreased in CC, leading to dysregulation of miR-196a and miR-205, which function as oncogenic miRNAs by targeting FOXO1 and PTEN, respectively. (**k**) DLX6 antisense RNA 1 (DLX6-AS1) may promote cell proliferation by sponging miR‑199a. (**l**) HOXD-AS1 upregulates *ZEB1* through binding to miR-130a-3p. (**m**) CRNDE acts as an oncogene in CC through sponging miR-183. (**n**) PVT1 acts as a sponge for miR-424, miR-195 and miR-200b. Overall, the figure shows some summarized molecular mechanisms by which the lncRNAs promote cell growth, migration and proliferation and EMT in a CC cell. Information extended in the text.

In CC, various studies have reported that lncRNAs are directly bound to target proteins or mRNAs to perform post-transcriptional modifications. Moreover, it has been suggested that lncRNAs play significant roles in CC progression by sponging miRNAs and interacting with HPV proteins [4,12]. In this context, we systematically summarized the dysregulation and the potential mechanisms of several lncRNAs such as Homeobox transcript antisense RNA (HOTAIR), H19, Metastasis-associated lung adenocarcinoma transcript 1 (MALAT1), Cervical Carcinoma High-Expressed 1 (CCHE1), Antisense noncoding RNA in the inhibitors of cyclin-dependent kinase 4 (ANRIL), Growth arrest special 5 (GAS5) and Plasmacytoma variant translocation 1 (PVT1), among others (Table 1, Figure 2), in cervical tumorigenesis.

### 3.1. HOTAIR

Homeobox transcript antisense RNA (HOTAIR), located at 12q13.13, is a 2.2 kb lncRNA derived from the *HOXC* gene cluster and is one of the most well-studied polycomb repressive complex 2 (PRC2)-interacting lncRNAs [86,87]. Interaction between HOTAIR and the polycomb repressive complex 2 (PRC2), which consists of SUZ12, EZH2, and EED, leads to the establishment of the repressive H3K27me3 chromatin mark resulting in transcriptional silencing through chromatin compaction [87,88].

Studies have found that HOTAIR functions as an oncogene in a great number of tumors including lung, hepatocellular, esophageal, ovarian, gastrointestinal, liver, breast cancer and others [15,89,90,91,92,93,94]. It has been revealed that increased expression of HOTAIR is associated with decreased survival rates in CC and may also be a potential therapeutic target because HOTAIR has a mechanistic role in the promotion of cell growth and invasion via modulation of the Notch/Wnt signaling pathway and the epithelial–mesenchymal transition (EMT) [16]. Additionally, in CC tissues and cell lines, HOTAIR expression was found to be significantly increased while the expressions of miR-143-3p and miR-23b were reduced [15,17]. HOTAIR knockdown suppresses proliferation and enhances apoptosis in CC cells. Moreover, HOTAIR could function as a sponge for miR-143-3p, promoting *BCL-2* expression. Overexpression of *BCL-2* reduces the tumor-suppressive effect of miR-143-3p in CC. HOTAIR overexpression is related to accelerated cell proliferation, CC cell growth and inhibited apoptosis (Figure 2a) [15].

It was demonstrated that HOTAIR knockdown can both inhibit cell proliferation and invasion and promote apoptosis. Furthermore, HOTAIR may indirectly modulate MAPK1 expression in CC cells by binding to miR-23b (Figure 2a). Nevertheless, it is necessary to understand the exact molecular mechanism to discover new potential approaches for early diagnosis and therapy, and the HOTAIR/miR-23b/MAPK1 axis could be a possible target in CC [17].

HOTAIR also exerts its tumor-promoting effect by sponging miR-17-5p (Figure 2a); therefore, the HOTAIR/miR-17-5p axis could also be a promising therapeutic target for future treatment of CC [18]. In addition, it was found that the mTOR/p70S6K pathway is activated by HOTAIR overexpression in CC, whose processes are involved in regulating cell growth, cell proliferation and cell survival by controlling mRNA translation, ribosome biogenesis and metabolism (Figure 2a) [19]. Correspondingly, a large number of studies found that an abnormal expression of HOTAIR was closely related to CC’s development, metastasis and invasion. HOTAIR expression also reflected the malignant development of CC, to some extent. Thus, the expression level of HOTAIR may have applications as a new biomarker in the diagnosis and treatment of CC [20].

### 3.2. H19

H19 is a 2.3 kb lncRNA encoded by the *H19* gene and is localized near the telomeric region of chromosome 11p15.5, within a unique locus shared with the *IGF2* gene. H19 is expressed exclusively from the maternal allele as a consequence of genomic imprinting. Alterations of gene expression at the *H19/IGF2* locus are associated with malignancies and developmental disorders [21,95,96,97]. Many studies have confirmed that H19 is associated with various tumors, such as gliomas, bladder, breast, pancreatic, esophageal, gastric cancer and others. H19 expression is closely related to tumor invasion, metastasis, recurrence and poor prognosis [98,99,100,101,102,103,104].

H19 RNA transcripts have been detected in a majority of patient-derived premalignant lesions of CC, being upregulated in CC lines and possibly secreted into extracellular vesicles. H19 promotes both anchorage-specific and independent growth of CC cell lines and may serve as a potential target for cancer diagnosis and therapy [105]. Additional studies showed that H19 interacts with various other miRNAs including the let-7 family (let-7a, let-7b, let-7c, let-7d, let-7e, let-7f, let-7g, let-7i, miR-98 and miR-202), miR-195, miR-138-5p and miR-106, acting as competitive endogenous RNA (ceRNA) and regulating diverse cellular processes in CC and others tumor types (Figure 2b). In normal physiological conditions, members of the let-7 family are mainly involved in gene regulation, cell adhesion, and muscle formation. Evidence suggests that let-7 is downregulated in several types of cancer [22,23,24]. Mir-195 acts as a tumor-suppressor in CC and it is downregulated in CC tissues [25]. MiR-138-5p is usually dysregulated in various types of cancer and is involved in proliferation, migration, invasion, angiogenesis and metabolism by targeting multiple mRNAs; it was shown that decreased expression of miR-138-5p in CC is related to malignant clinical features and poor prognosis of the disease [23]. Deregulation of miR-106 can promote proliferation and inhibit apoptosis of endometrial cancer RL95-2 cells; additionally, it is associated significantly with survival in human colorectal cancer patients [26,27].

Downregulation of H19 may have crucial functional importance in the cell, contributing to cervical carcinogenesis. It has been shown that H19 interacts with miR-675, miR-140, and miR-200, as well as sponge circular RNA (circRNA) MYLK, and binds competitively with miRNA-29a-3p to suppress CC [4].

Besides, H19 is modulated by the HPV16 E6 oncoprotein and might serve as an endogenous sponge for miR-138-5p in epithelial cells [22]. For all these findings, H19 may serve as a potential biomarker for CC prediction and may contribute to the treatment of the disease.

### 3.3. MALAT1

Metastasis-associated lung adenocarcinoma transcript 1 (MALAT1), also known as nuclear-enriched autosomal transcript 2 (NEAT2), located at 11q13, is an 8.5 kb lncRNA that is dynamically involved in several biological processes, such as epigenetic modification, alternative splicing, synapse formation and myogenesis. It has been demonstrated that MALAT1 contributes significantly to cancer development and progression [28,106,107].

MALAT1 presents differences in the degree of methylation in lung cancer tissues and normal tissues, demonstrating that its function as proto-oncogene can be regulated by methylation. Additionally, MALAT1 expression was significantly higher in patients with brain metastasis from lung cancer when compared to patients without brain metastasis [106].

This lncRNA participates in the modulation of various molecular signaling pathways, including MAPK/ERK, PI3K/AKT, WNT/β-catenin and NF-kB, leading to alterations in cell proliferation, cell death, cell cycle, migration, invasion, immunity, angiogenesis and tumorigenicity [28]. Overexpression of MALAT1 is positively correlated with tumor progression and metastasis in a large number of tumor types such as breast cancer, colon cancer and osteosarcoma [107,108,109].

It was showed that MALAT1 expression is significantly increased in CC than in normal tissues and is correlated with tumor size, FIGO stage, vascular invasion and lymph node metastasis [29]. MALAT1 expression is also upregulated in CC cell lines compared with normal cervical squamous cell samples and can promote cell migration and proliferation. Furthermore, HPV oncogenes correlate with MALAT1 deregulation in CC. MALAT1 may regulate cell proliferation through the P16^INK4A^/CDKs/RB pathway, but further studies are required to further elucidate this (Figure 2c) [30,31]. Downregulation of MALAT1 inhibits CC cell invasion and metastasis by inhibition of EMT [32]. Additionally, MALAT1 can modulate radiosensitivity of HR-HPV+ CC by sponging miR-145 [33]. Mir-145 acts as a tumor-suppressor targeting several genes and proteins. It regulates tumor growth, invasion and metastasis and participates in angiogenesis and tumor stem cell proliferation [34].

### 3.4. CCHE1

Cervical Carcinoma High-Expressed 1 (CCHE1) is a 2500 nt lncRNA located in an intergenic region on chromosome 10 (10q21.1). CCHE1 upregulation in CC is correlated with advanced FIGO stages, larger tumor size, lymph node metastasis, invasion of the uterine corpus, positive HPV and poor prognosis [35,38]. It was demonstrated that CCHE1 overexpression promotes the proliferation of CC cells; in contrast, its depletion inhibits the proliferation of CC cells. Furthermore, CCHE1 is physically associated with proliferating cell nuclear antigen (PCNA) messenger RNA, consequently enhancing the expression of PCNA (Figure 2d) [37]. Patients with low CCHE1 expression had a better overall survival and recurrence-free survival than patients with higher expression. Moreover, CCHE1 knockdown significantly promotes growth arrest and cell apoptosis through the activation of the ERK/MAPK pathway (Figure 2d) [38], suggesting that it is a potential prognostic biomarker for CC.

### 3.5. ANRIL

Antisense noncoding RNA in the inhibitors of cyclin-dependent kinase 4 (*INK4*) locus (ANRIL) is a 3.9 kb lncRNA located at the 9p21 region. The *INK4* locus encodes three tumor-suppressor genes: CDK inhibitors *p15* and *p16*, and *p14ARF* (a positive regulator of *p53*) [110]. It has been proved, since its identification, that ANRIL can deregulate several malignancies, such as gastric, breast, lung, bladder, prostate and nasopharyngeal cancers and multiple myeloma, usually functioning as a tumor-promoting lncRNA [111,112,113,114,115].

In CC, some studies reported that ANRIL is significantly upregulated and it is associated with advanced FIGO stage, lymph node metastasis and poor overall survival of patients with the disease. Additionally, ANRIL knockdown inhibits cell proliferation and metastasis in vitro, and its inhibition guides the inactivation of the PI3K/Akt pathway (Figure 1e). In CC tissues and CC cell lines, miR-186 is significantly downregulated by ANRIL when compared to adjacent noncancerous tissues and human epidermal cells; miR-186 suppresses cell proliferation and promotes apoptosis by downregulating Kazrin F. Thereby, the ANRIL/miR-186 axis might play a vital role in CC tumorigenesis. Downregulation of ANRIL may serve as a potential therapeutic target in the treatment of CC [39,40,41,42,43,114].

### 3.6. CCAT2

Colon cancer-associated transcript 2 (CCAT2), a 1752 bp lncRNA, located at 8q24.21, was originally detected as being highly expressed in colorectal cancer, promoting tumor process metastasis during carcinogenesis [44]. Recently, an increasing number of studies have shown that CCAT2 might play important roles in cancer progression. High CCAT2 expression is associated with the overall survival, progression-free survival lymph node and distant metastases of human cancers [45]. It was found that CC patients with high expression of CCAT2 have a shorter significantly overall survival. Knockdown of CCAT2 could induce CC cell cycle G1 phase arrest and may trigger apoptosis, promoting proliferation and survival of CC cells. These results strongly suggest that CCAT2 may be a potential prognostic factor and therapeutic target in patients with CC; however, the molecular mechanisms of CCAT2 that are involved in CC need to be further studied [44,45,46].

### 3.7. MEG3

Maternally expressed gene 3 (MEG3), is located at 14q32.3 within the DLK1-MEG3 locus. This gene contains ten exons and encodes around 1.6 kb long noncoding RNAs [116]. MEG3 is disrupted in several human cancers by epigenetic regulation—for instance, its expression can be modulated by DNA methylation. Additionally, its overexpression can promote apoptosis, suppress cell proliferation and impact the activity of the Wnt/β-catenin pathway in retinoblastoma [47,117]. Additionally, in lung cancer, it is involved in regulating the mark H3K27me3 for transcriptional repression [118]. In CC tissues, MEG3 is downregulated, compared to the adjacent normal tissues, and it is negatively related to FIGO stages, tumor size, HR-HPV infection, lymphatic metastasis and expression of miR-21. The level of miR-21-5p expression is reduced due to MEG3 overexpression, leading to inhibition of proliferation and increased apoptosis in CC cells. On the other hand, downregulation of MEG3 promotes the proliferation of CC cells and inhibits their apoptosis (Figure 2f). Therefore, it is suggested that MEG3 functions as a tumor-suppressor via regulating miR-21-5p, inhibiting the tumor growth in CC [47,48].

Additionally, MEG3 overexpression inhibits *Rac1* at both transcriptional and translational levels. *Rac1* encodes a GTPase belonging to the RAS superfamily of small GTP-binding proteins and regulates several cellular events in cancer, including cell growth, metastasis and cytoskeletal reorganization [49]. It was demonstrated that overexpression of MEG3 inhibits PI3K/AKT/BCL-2/Bax/P21 and PI3K/AKT/MMP-2/9 signaling pathways in Hela cells [50]. MEG3 also promotes P-STAT-3 degradation via ubiquitination, leading to inhibition of cell proliferation and affecting the development of CC [48]. For this reason, MEG3 may be considered as a potential biomarker and therapeutic target for CC.

### 3.8. BLACAT1

LncRNA bladder cancer-associated transcript 1 (BLACAT1, also known as linc-UBC1), located at 1q32.1 and with a length of 2616 bp, was initially identified and characterized in bladder cancer. However, studies have indicated that BLACAT1 is also overexpressed in other cancers, including gastric, lung and colorectal cancers [51,119]. BLACAT1 exerts a functional role in recruiting and binding to PRC2 and in promoting proliferation, migration and invasion by modulating Wnt/β-catenin signaling in CC (Figure 2e) [51,52,53]. Moreover, BLACAT1 is upregulated in cervical squamous cell carcinoma (CSCC) contributing to the migration and invasion of CSCC cells by downregulating miR-143 and miR-424 [54]. MiR-143 can play a role in CC pathogenesis by regulating the expression of BCL-2 [55]. In CC cell lines such as CaSki and SiHa, miR-424 expression inhibits cell growth which enhances apoptosis, blocks G1/S transition and suppresses cell migration and invasion [56]. Furthermore, knockdown of BLACAT1 in ME180 and C33A cells decreases the protein levels of cyclin B1, cell-division cycle 25C and N‑cadherin, while increasing the protein level of E‑cadherin [119]. For these reasons, BLACAT1 might be a potential novel prognostic biomarker for CC.

### 3.9. XIST

X-inactive specific transcript (XIST), located on the long arm of the human X chromosome (Xq13.2), was the first noncoding gene identified within the region of chromosome X inactivation center (XIC). XIST seems to play a potential role as a novel predictor of human cancer prognosis and presents an abnormal expression in various types of cancer [57,58]. The function of XIST and its impact on CC still needs to be identified but it can play the role of an oncogene, since its knocking down resulted in the repression of cell proliferation, invasion and migration by EMT via the Wnt/β-catenin signaling pathway (Figure 2h) [36]. It was found that XIST also contributes to CC’s tumor progression by inhibiting miR-140-5p that can act as an antitumorigenesis factor through targeting FEN1 mRNA. Moreover, a study explored the molecular mechanism of XIST/miR-140-5p/ORC1, providing a novel therapeutic method for CC [59].

### 3.10. SPRY4-IT1

SPRY4-Intronic transcript or Sprouty4-Intronic transcript 1 (SPRY4-IT1) is a 687 nt unspliced and polyadenylated transcript derived from the second intron of the SPRY4 gene, which is located at 5q31.3. It has recently been revealed as an oncogenic regulatory factor or tumor-suppressor in different types of human carcinomas, such as in melanoma, in which it is overexpressed [60,61,120]. Data indicate that SPRY4-IT1 is involved in cancer development through the regulation of alternative splicing or gene expression of its target genes. SPRY4-IT1 expression was upregulated in many solid tumors having a tumor-promoting function [62]. SPRY4-IT1 could directly bind to miR-101-3p (Figure 2i) and effectively act as ceRNA for miR-101-3p to regulate the expression of the target gene Zinc finger E-box binding homeobox 1 antisense 1 (ZEB1). MiR-101-3p acts as a tumor-suppressor in CC, and its overexpression can inhibit cell proliferation and invasion in vitro via regulating EMT, increasing the levels of E-cadherin and decreasing the levels of N-cadherin and vimentin in CC cells [61]. Thus, the SPYR4-IT1/miR-101-3p/ZEB1 axis contributes to CC migration and invasion, due to this, SPYR4-IT1 can be an important molecular biomarker for predicting prognosis and is a potential target for CC [60,63].

### 3.11. GAS5

Growth arrest special 5 (GAS5), located at 1q25.1, is a 650 nt lncRNA initially extracted from mouse NIH 3T3 cells using subtraction hybridization [64]. In various types of tumors, decreased expression of GAS5 is significantly associated with clinicopathological features such as TNM stage, histological grade, tumor size and distant metastasis [121]. When GAS5 is downregulated, it acts as a tumor-suppressor in several types of cancers, such as colorectal, breast, prostate, lung and ovarian cancers [65,122,123,124,125,126]. GAS5 expression is decreased in CC tissues and cells, and its overexpression suppresses CC cell proliferation, invasion and migration while inducing apoptosis of CC cells as well as suppressed tumor growth and metastasis by negatively regulating miR-196a and miR-205, which function as oncogenic miRNAs by targeting FOXO1 and PTEN, respectively (Figure 2j) [64,65]. Moreover, according to bioinformatics functional prediction, GAS5 acts as a molecular sponge for miR-106b which promotes cell proliferation and inhibits the apoptosis of endometrial cells [26,27,66].

Additionally, it was demonstrated that abnormal methylation of GAS5 contributes to poor expression of GAS5 in CC, suggesting that this lncRNA is a potential biomarker for this disease [67].

### 3.12. DLX6-AS1

DLX6 antisense RNA 1 (DLX6-AS1), is a 5.8 kb lncRNA located at the 7q21.3 region and is overexpressed in multiples carcinomas, including gastric cancer, breast cancer, lung cancer, gliomas and osteosarcomas [127,128,129]. In CC, it was found that DLX6-AS1 overexpression promotes tumoral progression by targeting the miR-16-5p/ARPP19 axis [68]. MiR-16-5p can modulate CC radiosensitivity through regulating coactivator-associated arginine methyltransferase 1. Furthermore, in prostate cancer, studies showed that this miRNA modulates the Cyclin D1/E1-pRb-E2F1 signaling pathway, enhancing the radiosensitivity of cancer cells [69].

High DLX6‑AS1 expression was associated with the FIGO stage and it was suggested that it may promote cell proliferation by serving as a ceRNA to sponge miR‑199a (Figure 2k). MiR-199a was significantly overexpressed in early CSCC and is presumed to promote the growth of CC cell lines. Additionally, its downregulation was significantly associated with lymph node metastasis and decreased prognosis survival in patients with advanced CSCC [70]. Knockdown of DLX6‑AS1 significantly induced cell apoptosis and inhibited cell growth in CC [71]. Additionally, DLX6-AS1 could enhance CC’s cell proliferation and invasion through upregulating FUS RNA binding protein [72]. This evidence suggests that DLX6-AS1 may be used as a prospective potential therapeutic target for CC treatment.

### 3.13. HOXD-AS1

HOXD antisense growth-associated long noncoding RNA (HOXD-AS1), also known as HAGLR or Mdgt, is transcribed from the HOXD cluster and its gene is located between the HOXD1 and HOXD3 genes at 2q31.1. It has been reported to play important roles in gut development and processes associated with cell apoptosis and metastasis in several cancers, including breast, liver, gastric and ovarian cancers [130,131,132,133,134]. HOXD-AS1 acts as a ceRNA to upregulate *ZEB1* through binding to miR-130a-3p in CC cells (Figure 2l) [73]. MiR-130a-3p controls cell growth, migration and invasion in a variety of cancer cells [74]. In CC tissues and cell lines, miR-130a-3p expression was significatively upregulated when compared with normal tissues and cells, promoting cell proliferation, migration and invasion, and inhibiting apoptosis [75]. When upregulated, HOXD-AS1 may work as an effective biomarker for CC via regulating the Ras/ERK pathway in vitro and in vivo. Therefore, HOXD-AS1 may serve as a new factor for predicting prognosis, as well as a promising therapeutic target for CC [76].

### 3.14. CRNDE

LncRNA colorectal neoplasia differentially expressed (CRNDE), located at 16q12.2, was initially identified as a lncRNA in colorectal cancer and reported to be highly elevated in this disease. In further studies, CRNDE was also upregulated in others malignant tumors such as hepatocellular, gastrointestinal, bladder, and others [135,136,137,138,139]. It was found that CRNDE is markedly upregulated in tissues and cell lines of CC. Its high expression is positively correlated with advanced FIGO stage, lymph node metastasis and poor overall survival rate. Furthermore, it was demonstrated that CRNDE influences proliferation and apoptosis in CC cells by targeting PI3K/AKT pathway (Figure 2m) [77]. Other studies showed that CRNDE acts as an oncogene in CC through sponging miR-183 (Figure 2m) to regulate CCNB1 expression, so, the role of CRNDE/miR-183/CCNB1 axis offers a promising diagnostic strategy for CC treatment [78]. CRNDE also plays an oncogenic effect on the progression of CC by suppressing p53 upregulated modulator of apoptosis (PUMA) expression and reducing the PUMA signaling pathway, suggesting that this axis might be a potential target for developing effective therapeutic strategies to prevent CC progression [79].

### 3.15. PVT1

Plasmacytoma variant translocation 1 (PVT1) is a 1716 nt lncRNA located at 8q24.21. This chromosomal region is known as a gene desert, because of its lack of protein-coding genes. It contains a large number of risk alleles that are implicated in cancer [140,141]. Elevated PVT1 expression was related to poor prognosis and might be a potential biomarker of clinicopathological characteristics in different cancer types, such as gastric, ovarian, breast cancers, and others [142,143,144,145]. In CC, studies revealed that PVT1 acts as a sponge or ceRNA for miR-424 (Figure 2n), this miRNA may act as a novel tumor suppressor in CC by blocking cell growth through targeting the KDM5B-Notch pathway [80]. PVT1 can also promote proliferation and metastasis by increasing the Smad3 expression through sponging miR-140-5p, which, as mentioned above, may act as an antitumorigenesis factor targeting FEN1 mRNA [59,81]. PVT1 also can bind directly to EZH2, increasing H3K27me3 level on miR-195 and miR-200b promoters, inhibiting their expression (Figure 2n). MiR-195 is associated with EMT and chemoresistance and miR-200b with cell proliferation, invasion and migration of CC cells. Furthermore, knockdown of HPV16 E7 significantly inhibited PVT1 and restored miR-195 expression [82,83]. Likewise, PVT1 can promote the growth of HPV positive and negative CSCC by inhibiting TGF-β1 [84]. It was observed that serum PVT1 level is mostly increased in CC patients and correlated with tumor size, FIGO stage and lymph node metastasis, demonstrating that PVT1 may be a novel noninvasive biomarker for early diagnosis of CC [85].

### 3.16. Others LncRNAs in CC

Along with the above-mentioned lncRNAs (Table 1), many other novel factors have been reported to be dysregulated in CC and can play multiples roles in its promotion and progression, nevertheless, further studies are necessary.

Cancer susceptibility candidate 11 (CASC11) is a lncRNA that is positively associated with tumor size and FIGO staging, and negatively related to the patients’ survival rates. It was demonstrated that this lncRNA is upregulated in the CC tissues and cell lines, and its silencing inhibits proliferation, migration as well as invasion and promoted cell apoptosis. CASC11 promotes CC progression by activating the Wnt/βcatenin signaling pathway [146].

Tumor Protein P73 Antisense RNA 1 (TP73-AS1) is a lncRNA that promotes cancer progression in several types of cancers. It is upregulated in CC cells, inhibiting miR-329-3p expression, while miR-329-3p inhibits ADP Ribosylation Factor 1 (ARF1) expression [147]. Furthermore, upregulation of TP73‑AS1 promotes CC progression by promoting CCND2 through the suppression of miR‑607 expression [148].

Tumor protein translationally controlled 1 (TPT1) antisense RNA 1 (TPT1-AS1) is a lncRNA that is upregulated in CC, promoting cell growth and metastasis by acting as a ceRNA for miR-324-5p [149]. The functional diversity of TPT1-AS1 in various malignancies may be caused by tumor heterogeneity, and further investigations are necessary to reveal the potential roles of this lncRNA [150].

Zinc finger E-box binding homeobox 1 antisense 1 (ZEB1-AS1), is a cancer-related lncRNA that acts as an oncogenic regulator in diverse types of cancer. In CC, ZEB1-AS1 promotes cell invasion and epithelial to mesenchymal transition through inducing ZEB1 expression [151].

Myocardial infarction associated transcript (MIAT) is another lncRNA that is involved in PI3K/AKT signaling pathway suppression, promoting cell proliferation and invasion in CC [152].

Another study identified six lncRNAs (TMEM220-AS1, TRAM2-AS1, C5orf66-AS1, RASSF8-AS1, AC126474 and AC004908), which might play important roles in tumor progression of CC. Two of them might be associated with the prognosis of the disease, providing new insights into the diagnosis and treatment of CC. This analysis showed that these hub lncRNAs are involved in keratinization-related pathways, as well as immune-related pathways, implying the important role of these two pathways in regulating CC progression [153].

## 4. Perspectives and Challenges

The current studies and research on the functions and levels of the lncRNAs in CC open up new possibilities for the evaluation of molecular markers for the disease.

However, even though the ability of lncRNAs to sponge specific miRNAs and to deregulate metabolic pathways has been investigated in cell culture models, tissues and CC cells, the biggest challenge regarding working with lncRNAs is the comprehension of the molecular mechanisms underlying their functions as well as their correlation with others ncRNAs (such as mRNAs, miRNAs, and circRNAs) and proteins, which is still not fully resolved. In the same way, lncRNAs’ interactions with the immune system, microenvironment, microbiome, hormonal milieu, or metabolome is not completely elucidated either.

Overall, we summarized the mains aspects of lncRNA-mediated epigenetic regulation, highlighting that the lncRNAs mentioned in this review have great potential to be applied as biomarkers for the prognosis and metastasis in CC, which currently remains a public health issue and one of the leading causes of women’s death worldwide. Studies concerning the association of lncRNAs in CC are still in their early stages, but recent evidence points towards the possible use of these ncRNAs as key molecular tools in understanding the disease. Therefore, more experimental and clinical studies are necessary for the exploration and characterization of the mechanisms by which the lncRNAs are involved in the prognosis, invasion, metastasis, chemoresistance and radioresistance in CC, offering novel alternative approaches for better diagnosis and therapy in the future.

## Figures and Tables

**Table 1:** lncRNAs and their mechanisms and biological roles in Cervical cancer (CC).

LncRNA	Localization	Expression Level	Signaling Pathways and Molecules	Biological Roles	Ref.
HOTAIR	12q13.13	Upregulated	Notch/Wnt pathway, sponge for miR-143-3p, miR-23b and miR-17-5p. MAPK1, BCL-2, mTOR/p70S6K pathway.	Cell growth, cell proliferation and cell survival, EMT metastasis, and invasion	[15,16,17,18,19,20]
H19	11p15.5	Upregulated	Sponge for let-7 family, miR-106, miR-194, miR-138-5p, miR-675, miR-140, miR-200 and MILK	Cell migration, cell proliferation	[21,22,23,24,25,26,27]
MALAT1	11q13	Upregulated	P16^INK4A^/CDKs/RB pathway, sponge for miR-145.	Cell migration, cell proliferation, vascular invasion, EMT, metastasis.	[28,29,30,31,32,33,34]
CCHE1	10q21.1	Upregulated	ERK/MAPK pathway. PCNA.	Cell proliferation, metastasis.	[35,36,37,38]
ANRIL	9p21	Upregulated	PI3K/Akt pathway, Sponge for miR-186.	Cell proliferation, metastasis	[39,40,41,42,43]
CCAT2	8q24.21	Upregulated	Cell cycle	Cell proliferation, metastasis.	[44,45,46]
MEG3	14q32.3	Downregulated	Sponge for miR-21-5p. PI3K/AKT/BCL-2/Bax/P21 pathway, PI3K/AKT/MMP-2/9 pathway, P-STAT3.	Metastasis, apoptosis.	[47,48,49,50]
BLACAT1	1q32.1	Upregulated	Wnt/β-catenin pathway, sponge for miR-143 and miR-424.	Cell proliferation, cell migration.	[51,52,53,54,55,56]
XIST	Xq13.2	Upregulated	Wnt/β-catenin pathway, sponge for miR-140-5p.	Cell proliferation, cell invasion, cell EMT, migration.	[36,57,58,59]
SPRY4-IT1	5q31.3	Upregulated	sponge for miR-101-3p. ZEB1.	Cell proliferation, cell migration	[60,61,62,63]
GAS5	1q25.1	Downregulated	Sponge for miR-196a and miR-205.	Cell proliferation, cell invasion, apoptosis,	[64,65,66,67]
DLX6-AS1	7q21.3	Upregulated	Sponge for miR-16-5p and miR-199a. ARPP19, FUS.	Cell proliferation, cell invasion.	[68,69,70,71,72]
HOXD-AS1	2q31.1	Upregulated	Ras/ERK pathway, sponge for miR-130a-3p. ZEB1.	Cell growth, cell migration, cell invasion.	[73,74,75,76]
CRNDE	16q12.2	Upregulated	PI3K/AKT pathway, sponge for miR-183. CCNB1, PUMA, P53.	Cell proliferation, apoptosis.	[77,78,79]
PVT1	8q24.21	Upregulated	Smad3, sponge for miR-140-5p, miR-424, miR-195 and miR-200b. EZH2, TGF-β1.	Cell growth, cell proliferation, metastasis.	[80,81,82,83,84,85]

## Abbreviations

CC	Cervical Cancer
ncRNA	Noncoding RNAs
lncRNA	Long noncoding RNA
nt	Nucleotides
HPV	Human Papillomavirus
HR-HPV	High-Risk Human Papillomavirus
SCC	Squamous Cell Carcinoma
CSCC	Cervical Squamous Cell Carcinoma
mRNA	Messenger RNA
rRNA	Ribosomal RNA
tRNA	Transfer RNA
snRNA	Small Nuclear RNA
snoRNA	Small Nucleolar RNA
miRNA	MicroRNA
siRNA	Small Interfering RNA
piRNA	Piwi-interacting RNA
circRNA	Circular RNA
ceRNA	Competitive Endogenous RNA
EMT	Epithelial–Mesenchymal Transition
